# Education Research: Monitoring and Tracking Neurophobia: Evidence From a Temporal Analysis of Brazilian Medical Schools

**DOI:** 10.1212/NE9.0000000000200076

**Published:** 2023-06-09

**Authors:** Arthur N. Rodrigues, Tarsis S. Sousa, Márcio C.R. Marvão, Diego S. Sena, Brenda H.B. Koshimoto, Serginara C.F.P. Silva, Vitoria V.C. Monteiro, Ana Luisa R. Fraiha, Renato C. Santos, Bruno L. Santos-Lobato

**Affiliations:** From the Centro de Ciências Biológicas e da Saúde (A.N.R., T.S.S., B.L.S.-L.), Universidade do Estado do Pará; Instituto de Ciências Médicas (M.C.R.M., D.S.S., B.H.B.K.), Universidade Federal do Pará; Centro Universitário do Pará (S.C.F.P.S., V.V.C.M.); and Centro Universitário Metropolitano da Amazônia (A.L.R.F., R.C.S., B.L.S.-L.), Belém, Brasil.

## Abstract

**Background and Objectives:**

Neurologic disorders are common medical conditions. However, even with a higher demand for neurologic care, the capacity to train neurologists is impaired. The fear of neurosciences/neurology by medical students, known as neurophobia, may cause multiple adverse effects in neurologic assistance. The objectives of this study were to estimate the current prevalence and characteristics of neurophobia in medical students in Brazil and to compare neurophobic symptoms at 2 time points.

**Methods:**

This is a cross-sectional study conducted with students from 4 medical schools in Pará, Brazil, who matriculated into the preclinical stage, clinical stage, and internship were submitted to a questionnaire to assess the perception of clinical specialties, including neurology. Reasons for neurophobia, probable strategies to improve neurologic education, and a specific scale to detect neurophobia were also evaluated. Furthermore, we performed a temporal comparison of current results with those from a previous study from 2015.

**Results:**

Neurophobia was detected in 63.3% of medical students. The perception of the difficulty in neurology/neurosciences was very high in all stages, and the perception of interest and quality of teaching worsened during the internship. The need to understand neuroanatomy and neurophysiology was cited as the most important reason for neurophobia. More and better bedside tutorials were the most mentioned suggestion to improve neurologic education. The temporal comparison between 2015 and 2022 showed that the level of knowledge, quality of teaching, and likelihood of pursuing a career in neurology has become more favorable in 2022.

**Discussion:**

The prevalence of neurophobia in Brazil was higher than that in high-income countries. Unfavorable opinions about neurology tended to increase throughout the medical course, but the temporal comparison showed that the impact of neurophobia has decreased. Surveillance systems for monitoring and tracking neurophobia should be implemented in medical schools.

Neurology is a clinical specialty that assists many typical medical conditions, such as headache, epilepsy, and stroke,^1^ as well as neurodegenerative diseases with increasing prevalence, such as Alzheimer disease and Parkinson disease.^1^ Thus, there is a strong demand for medical workers to provide neurologic care.

However, the world is struggling to increase its neurologic workforce. Even developed countries, such as the United States, report that the current supply of neurologists is insufficient to assist with the burden caused by nervous system diseases.^2,3^ In Brazil, there is a lack of neurologic workforce in poorer regions and a low number of programs qualified to train new neurologists.^4^

Beyond the low capacity to train new neurologists, medical students worldwide have complained about a fear of neurosciences and neurology, called neurophobia.^5^ Neurophobia is associated with unfavorable opinions about neurology, mainly related to a supposed inherent difficulty in understanding the theme, a complicated physical examination, and caring for devastating diseases without effective treatments.^6^ According to students from different backgrounds, the need to understand neuroanatomy and neurophysiology is commonly cited as the main reason for neurophobia,^6-11^ and better integration between neurosciences and clinical neurology would be the most efficient way to mitigate the problem.^5^

Besides being exclusively an educational issue, neurophobia is also a public health problem. It reduces the number of future neurologists, harms general physicians' knowledge about the management of common neurologic diseases, and can promote issues in referring patients to neurologists.^3^ All these factors preclude adequate neurologic assistance in health systems.

Thus, the monitoring of neurophobia and the application of new educational approaches to mitigate neurophobic symptoms are important for the quality of neurologic care. In 2018, our group reported the first data about neurophobia in Brazil, confirming that Brazilian medical students had unfavorable opinions about neuroscience and neurology.^6^ Since then, there has been an advance of active learning methods in medical schools, the awareness about neurophobia has improved, and the coronavirus disease 2019 (COVID-19) pandemic has affected global medical education. Our understanding of neurophobia in Brazil may support strategic measures in similar developing countries. This study aimed to reevaluate neurophobia in Brazilian medical students, comparing the results with those of our first study.

## Methods

### Study Design and Sample

We conducted a cross-sectional study to estimate (1) the prevalence of neurophobia in medical students, as well as (2) the perception of neurology/neurosciences, possible reasons for neurophobia, and suggestions to improve neurology/neurosciences teaching and learning. We also (3) compared the current results with those from a previous study from 2015.

The study recruited participants from July to September 2022 at 4 medical schools in the state of Pará, Northern Brazil: Universidade Federal do Pará, Universidade do Estado do Pará, Centro Universitário do Pará, and Centro Universitário Metropolitano da Amazônia. Medical schools in Brazil adopt a course with 6 years of training (years 1 and 2: preclinical stage; years 3 and 4: clinical stage; and years 5 and 6: internship stage). Based on their current medical curriculum, there are differences regarding neurologic teaching: 3 of these medical schools use active learning strategies and provide multiple exposures to neural sciences, neurologic semiology, and clinical neurology throughout preclinical and clinical stages; one of these medical schools uses a mixed approach with active learning techniques and traditional lectures and provides lesser exposure to neurology.

As inclusion criteria, we included only medical students from year 2, year 4, and year 6 who matriculated into the cited medical schools. A total of 1,643 medical students were matriculated in years 2, 4, and 6 of these medical schools (year 2: 561; year 4: 538; and year 6: 544) in 2022. We opted to enroll only medical students from these years due to higher exposure to neurosciences/neurology in each medical school stage and to optimize the recruitment process.

In addition, we performed an open online survey with medical students using the same evaluation instrument converted into an online form. Considering the convenience of performing evaluations with this method, we included students without the restriction of medical school or year of training.

To compare neurophobia in 2 time points, we performed a post hoc temporal analysis of the current results and raw data from Santos-Lobato et al.^6^ Our group conducted this previous survey in 2015. It used a similar methodology to assess the perceptions, reasons, and suggestions related to neurophobia in 3 of the 4 medical schools evaluated in this study.

### Assessment and Outcome Measures

We recorded demographic data (age at evaluation, sex, medical school year, and enrollment in extracurricular activities). For the assessment of neurophobia, we used a semistructured questionnaire previously described.^6^ In brief, the first section assessed the perception of 7 clinical specialties (cardiology, endocrinology, gastroenterology, nephrology, neurology, respiratory medicine, and rheumatology) according to 6 categories (level of interest, level of knowledge, degree of difficulty, confidence in examining patients, quality of teaching at medical school, and likelihood of pursuing a career in the specialty). The responses were ranked on a Likert scale of 5 items.

In the second section, participants graduated the importance of selected reasons for neurology being perceived as a difficult subject and the usefulness of selected suggestions to improve neurologic education. The responses were ranked on a Likert scale of 4 items. Participants could also answer an open-ended question about how teaching in neurology/neurosciences could be improved.

Finally, we added a recently described scale to the questionnaire, constructed to measure and detect neurophobia, the NeuroQ.^12^ The NeuroQ is a 5-item scale, and each item scores from 1 to 5, with a minimum value of 5 and a maximum of 25, suitable for medical students in preclinical years. The scale was already validated in medical students from France.^12^ A NeuroQ score above 16 defines a student with neurophobia, and a NeuroQ score above 18 indicates marked neurophobia. We translated the questions from English to Portuguese (eTable 1, links.lww.com/NE9/A32).

For in-person evaluations, the questionnaires were distributed to the medical students who accepted participating in the survey after their regular lectures. The questionnaires were written and self-administered, and students were asked not to indicate their names. For online evaluations, a Google Forms–based questionnaire was shared through social media for medical students. Questionnaires with less than 50% of questions answered were excluded.

The primary outcome was the prevalence of neurophobia and marked neurophobia, according to NeuroQ. Key secondary outcomes were NeuroQ score, the perception of neurology/neurosciences by category, the opinions about reasons for neurology/neurosciences being perceived as difficult subjects, and suggestions to improve neurology/neurosciences teaching and learning.

The temporal analysis of neurophobia was performed through the comparison of the perception of neurology/neurosciences, reasons for neurology being perceived as a difficult subject, and the usefulness of selected suggestions to improve neurologic education of this study (2022) compared with the results of our previous survey from 2015.^6^ An improvement or worsening of perceptions, reasons, and suggestions about neurology/neurosciences from 2015 to 2022 was defined based on a significant increase or reduction in the number of favorable opinions.

### Statistical Analysis

For sample size calculation, under the assumption of a margin of error of 5%, with a confidence level of 99% and a sample proportion of 50%, a minimum of 473 participants in years 2, 4, and 6 (estimated population of 1,643 medical students) would be needed. We performed the χ^2^ test for categorical variables. For continuous variables, differences between 2 independent samples were calculated using the Mann-Whitney test. Differences between 3 or more independent samples were calculated using the Kruskal-Wallis test and the Dunn procedures for multiple comparisons. For the temporal analysis of neurophobia, the differences between Likert-based responses about the perceptions, reasons, and suggestions regarding neurology/neurosciences of this study and those of the previous survey were calculated using the χ^2^ test. Missing data were not imputed. All analyses were performed using SPSS for Windows version 23.0 (SPSS Inc., Chicago, IL). The textual analysis of the open-ended question answers was performed with the open-access text analysis software Interface de R pour les Analyses Multidimensionnelles de Textes et de Questionnaires version 0.7 alpha 2, through the word cloud method.^13^

### Standard Protocol Approvals, Registrations, and Patient Consents

The study was approved by the Universidade do Estado do Pará Ethics Committee (CAAE 52785521.5.0000.5174). All institutions approved the study. All participants provided written informed consent. We used the Strengthening the Reporting of Observational Studies in Epidemiology cross-sectional reporting guideline checklist when writing our report.^14^

### Data Availability

Anonymized data not published within this article will be made available by request from any qualified investigator.

## Results

### Epidemiologic Data of the Sample

A total of 827 questionnaires were filled, 639 filled in person (77.3%) and 188 (22.7%) online. The proportion of participants through in-person questionnaires represented 38.8% of all students matriculated in years 2, 4, and 6 from 4 medical schools (year 2: 32.6%; year 4: 49.8%; and year 6: 34%).

Three in-person questionnaires were excluded because of filling less than 50% of the questions (response rate: 99.6%). Data are summarized in Table 1. Regarding sex, there was an equal distribution among medical students. There were no relevant differences between answers from in-person and online questionnaires.

**Table 1:**
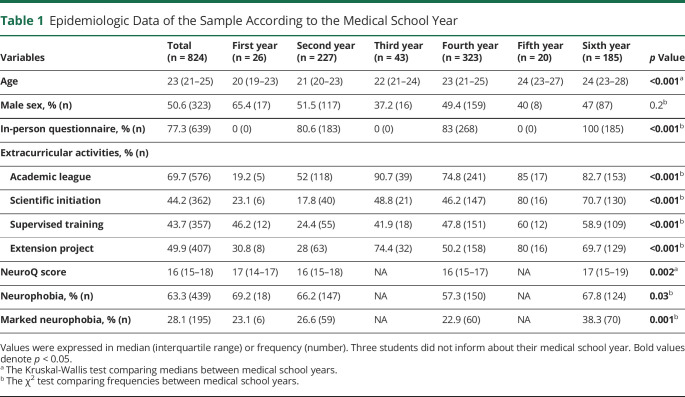
Epidemiologic Data of the Sample According to the Medical School Year

### Diagnosis of Neurophobia According to the NeuroQ Questionnaire

A total of 693 medical students answered the NeuroQ questionnaire (preclinical stage: n = 248; clinical stage: n = 262; and internship: n = 183). The median score of NeuroQ was 16 points (interquartile range 15–18), being higher in the internship than in the clinical stage (Kruskal-Wallis statistics = 12, *p* = 0.002; clinical vs internship, *p* = 0.002) (eFigure 1, links.lww.com/NE9/A31). Neurophobia was diagnosed in 63.3% of the sample (preclinical stage 66.5%, clinical stage 57.3%, and internship 67.8%) and marked neurophobia in 28.1% (preclinical stage 26.2%, clinical stage 22.9%, and internship 38.3%). The internship was the medical school stage with higher frequencies of neurophobia (χ^2^ statistics = 6.81, *p* = 0.03) and marked neurophobia (χ^2^ statistics = 13.2, *p* = 0.001) (Table 1). There was no difference between male and female students according to the NeuroQ questionnaire.

### Perception of Neurology and Neurosciences Among Medical Students

As summarized in Table 2, there was no apparent modification of the perception of neurology/neurosciences' degree of difficulty throughout the medical training (χ^2^ statistics = 10, *p* = 0.26). Perceived level of interest (χ^2^ statistics = 26.1, *p* = 0.001) and quality of teaching of neurology/neurosciences (χ^2^ statistics = 207.1, *p* < 0.001) decreased along with the medical course, but there was an increase in the perception of knowledge (χ^2^ statistics = 35.5, *p* < 0.001) in the last years. The choice of neurology as the first option for a future career decreased in the clinical and internship stages compared with that in the preclinical stage. The rejection of neurology (seventh option) increased in the late medical stages. Neurology was the first option for a future career for 20.5% of medical students and the last option for 25.2%.

**Table 2:**
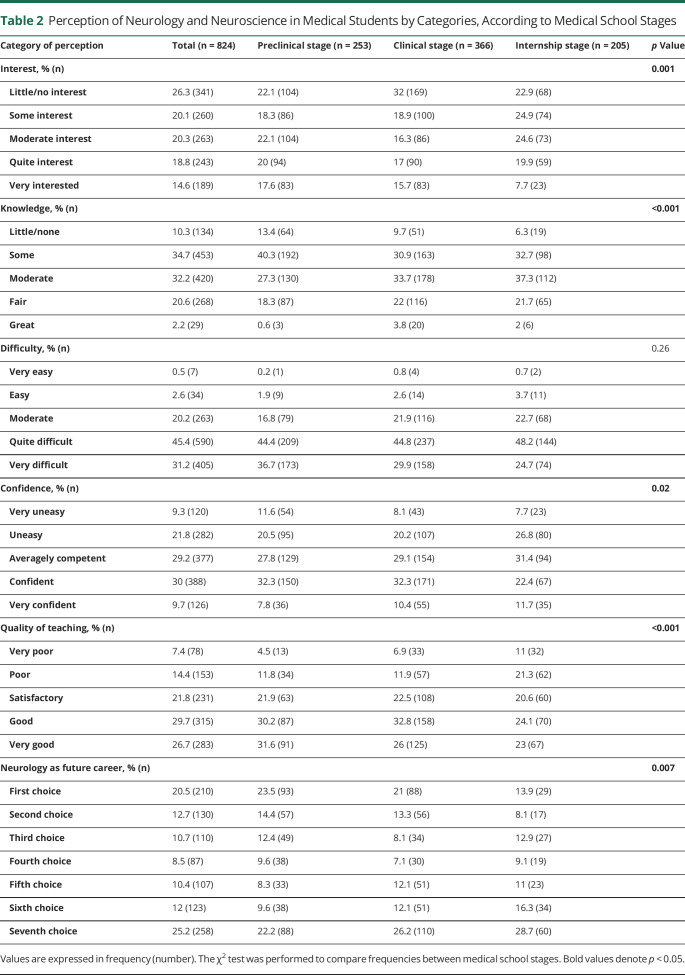
Perception of Neurology and Neuroscience in Medical Students by Categories, According to Medical School Stages

### Reasons for Neurology and Neurosciences Being Perceived as Difficult Subjects

The need to understand neuroanatomy and neurophysiology was cited as the most important reason for neurophobia in all medical school stages. However, some reasons were more relevant for internship students than preclinical students (not having enough teaching time, an important and a very important factor for neurophobia—preclinical: 46%, internship: 60%; having a complex examination, an important and a very important factor for neurophobia—pre-clinical: 63%, internship: 76.1%; being poorly taught, an important and a very important factor for neurophobia—preclinical: 42%, internship: 54.9%) (Table 3).

**Table 3:**
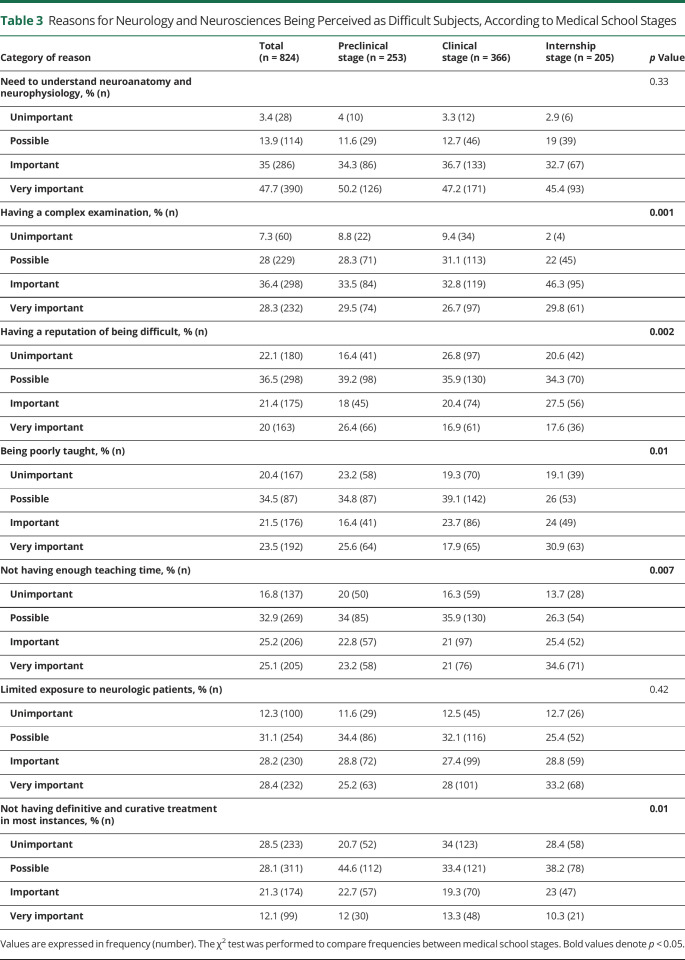
Reasons for Neurology and Neurosciences Being Perceived as Difficult Subjects, According to Medical School Stages

### Suggestions to Improve Neurology and Neurosciences Teaching and Learning

More and better bedside tutorials were the most cited suggestion to improve neurology/neurosciences teaching and learning (very useful and extremely useful change to improve teaching—pre-clinical: 86.5%, clinical: 89.8%, and internship: 92.3%; χ^2^ statistics = 14, *p* = 0.028). The medical school stage did not significantly affect the suggestions to improve neurology/neurosciences teaching and learning. Furthermore, internship students had more unfavorable opinions about the role of textbooks than preclinical students (Table 4).

**Table 4:**
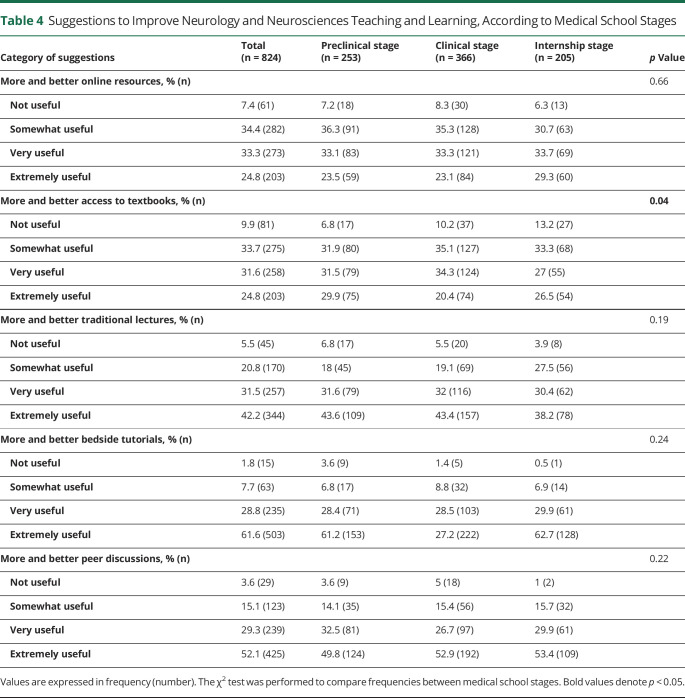
Suggestions to Improve Neurology and Neurosciences Teaching and Learning, According to Medical School Stages

Regarding the open-ended question about how teaching in neurology/neurosciences could be improved, the word cloud method of 437 answers confirmed the relevance of practical scenarios for students. The top 5 most cited words were as follows: “lecture” (247 citations), “practical” (212 citations), “clinical” (121 citations), “case” (92 citations), and “patient” (82 citations). Most answers addressed particular issues from each medical school. To increase the time on practical training, some students proposed a flipped classroom–based strategy, with lectures through video at home. In addition, some students suggested that the neurosciences teaching by neurologists in the preclinical stage would be better than by non-neurologists.

### Temporal Comparison of Neurophobia

**Figure 1:**
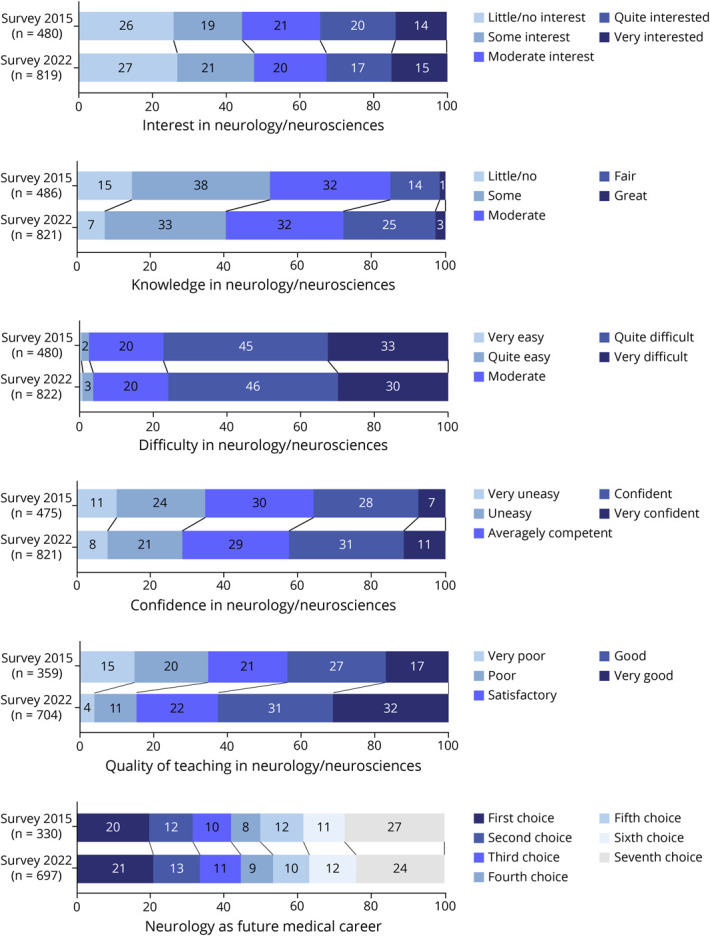
Temporal Comparison of the Perception of Neurology and Neuroscience in Medical Students by Categories Between 2015 and 2022 Numbers in stacked bars indicate the percentage of responses ranked on a Likert scale of 5 items (except the category “neurology as future medical career” with 7 items).

The perception of neurology/neurosciences was more favorable in 2022 compared with that in 2015 in the level of knowledge (χ^2^ statistics = 216, *p* < 0.001), quality of teaching (χ^2^ statistics = 136, *p* < 0.001), and the likelihood of pursuing a career (χ^2^ statistics = 260, *p* < 0.001) (Figure 1, eFigure 2, links.lww.com/NE9/A31).

**Figure 2:**
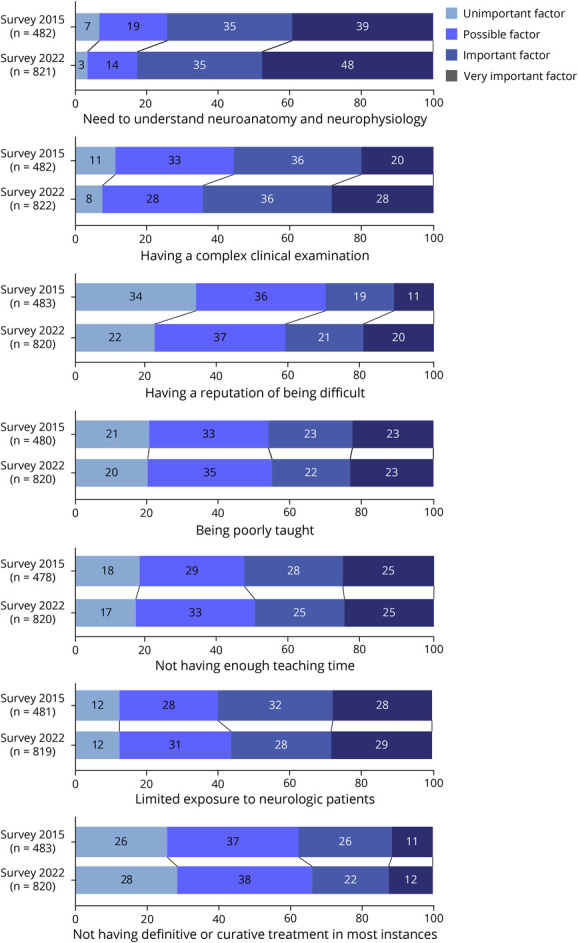
Temporal Comparison of the Reasons for Neurology and Neurosciences Being Perceived as Difficult Subjects Between 2015 and 2022 Numbers in stacked bars indicate the percentage of responses ranked on a Likert scale of 4 items.

As in 2015, the need to understand neuroanatomy and neurophysiology was the most cited reason for neurophobia, with an increase in the proportion of answers (an important and a very important factor for neurophobia—2015: 74.2%; 2022: 82.4%; χ^2^ statistics = 16.5, *p* < 0.001). The complex clinical examination became the second most cited reason for neurophobia in 2022 (an important and a very important factor for neurophobia—2015: 55.8%; 2022: 64.6%; χ^2^ statistics = 15.6, *p* < 0.001). Previously, limited exposure to neurologic patients was the second most cited reason for neurophobia (an important and a very important factor for neurophobia—2015: 60%; 2022: 56.4%; χ^2^ statistics = 2.9, *p* = 0.4). There was a worsening in the reputation of neurology as a difficult subject in 2022 (an important and a very important factor for neurophobia—2015: 30.2%; 2022: 41.4%; χ^2^ statistics = 30.2, *p* < 0.001) (Figure 2).

**Figure 3:**
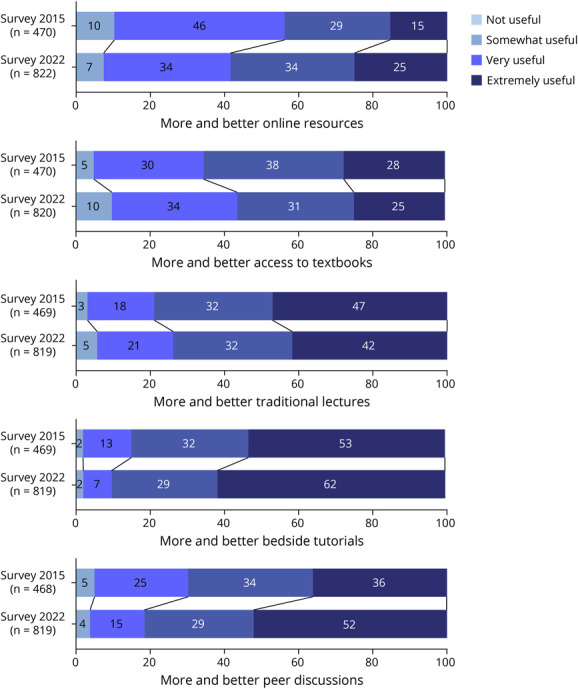
Temporal Comparison of the Suggestions to Improve Neurology and Neurosciences Teaching and Learning Between 2015 and 2022 Numbers in stacked bars indicate the percentage of responses ranked on a Likert scale of 4 items.

There was an increase in answers suggesting more and better peer discussions in 2022 than in the former survey (a very useful and an extremely useful change to improve teaching—2015: 69.6%; 2022: 81.4%; χ^2^ statistics = 37.4, *p* < 0.001). While more and better access to textbooks was less suggested in 2022 (a very useful and an extremely useful change to improve teaching—2015: 65.3%; 2022: 56.5%; χ^2^ statistics = 14.8, *p* = 0.002), the relevance of online resources to improve neurology/neurosciences teaching increased (a very useful and an extremely useful change to improve teaching—2015: 43.8%; 2022: 58.3%; χ^2^ statistics = 28.2, *p* < 0.001) (Figure 3).

## Discussion

In this cross-sectional study in Pará, Brazil, approximately two-thirds of our medical students (63.3%) were diagnosed with neurophobia, which is more severe in 28% of our sample. The prevalence of neurophobia was higher in the internship stage. In addition, the perception of neurology/neurosciences in the last years of medical school worsens, according to the level of interest, quality of teaching, and the likelihood of pursuing a career in neurology. neurology/neurosciences was perceived as the most difficult clinical subject, regardless of medical stage. The need to understand neuroanatomy and neurophysiology was the most cited reason for neurophobia for all medical stages. The medical students suggested that a methodology strongly based on bedside teaching would be an extremely useful change to improve neurology/neurosciences teaching and learning.

Before the NeuroQ score, there was no specific tool to measure the prevalence of neurophobia, also suitable for preclinical medical students. A previous study in Singapore reported a prevalence of 47.5% of neurophobic students.^15^ The original study from France, which described the NeuroQ score, showed that 25.1% of medical students had neurophobia and 7.3% marked neurophobia.^12^ In his seminal article, Jozefowicz^5^ suggested that half of medical students experience neurophobia at 1 point during medical training. Comparing our results with these cited studies, the prevalence of neurophobia found in Pará was 2-fold higher than in regions located in high-income countries.

Neurophobia is present in developing countries, with similar perceptions and reasons as seen in developed regions.^6,8,10,11^ However, there are no data about the prevalence of neurophobia in other developing countries to explore a possible association between socioeconomic conditions and neurophobia. A previous study showed that the individual socioeconomic background, such as debt level, household income, and parental education level, may affect the interest in brain-related specialties.^16^ It is reasonable to suggest that vulnerable socioeconomic conditions in students before medical school and low-resource settings may promote fear of neurosciences and neurology.

For Brazil, the high prevalence of neurophobia may be partially explained by the lack of a core curriculum in basic neurosciences and clinical neurology and a shortage of neurologists in poorer regions.^4^ In a global context, the present results are relevant for other developing countries, particularly on the prevalence of neurophobic symptoms, the worsening of opinions about neurology in the internship, and the effect of mitigating measures on neurophobia.

Classically, neurophobia occurs mainly during the neural science course in the preclinical stage and the internship.^5^ Unfortunately, because most studies excluded preclinical medical students, the presence of neurophobia in all medical school stages was less explored. Fantaneanu et al.^17^ compared the perception of medical schools at the medical school stage with a “wearing-off effect” with more unfavorable opinions about neurology/neurosciences in the preclinical years, followed by a more favorable perception in clinical years, but with a worsening of neurophobia in the internship (called “neuramnesia”), as a U-shaped curve. Our study found this U-shaped pattern in the prevalence of neurophobia and marked neurophobia. However, our medical students in the internship stage tended to have the most unfavorable opinions about neurology/neurosciences.

In the internship stage, students reported that the level of knowledge, confidence in examining patients, and quality of teaching at medical school for all other evaluated clinical specialties increased (eTable 2, links.lww.com/NE9/A33). The level of interest for all other clinical specialties in the internship stage also increased, except for Cardiology (eTable 2). These results highlight the uniqueness of unfavorable opinions about neurology in the last years of medical school. It has been proposed that the students' perceptions of neurologists as unhappy physicians and longer gaps between neurologic teaching throughout the medical course may be potential reasons for neurophobia in internship students.^17^

The need to understand neuroanatomy and neurophysiology and having a complex examination were the most commonly cited reason for neurophobia in our study. The first reason was cited as the main reason for neurophobia in previous studies.^6-11^ According to a classification of reasons for neurophobia, the need to understand neuroanatomy and neurophysiology can be linked to “preconceptions” and “barriers to learning” themes associated with neurophobia. The “preconception” theme may be a nonmodifiable risk factor of neurophobia, but the “barriers to learning” theme may be modified through methodological adaptations.^17^

Effective integration between basic neurosciences and clinical neurology is the most important treatment for neurophobia.^5^ However, the task is not easy, considering that neuroanatomy is perceived as the most difficult theme compared with other anatomy topics.^18^ In addition, most neuroanatomy lecturers may overestimate the relevance of the discipline in the medical curriculum and deny the need for changes in their teaching and learning process.^19^ Although teaching and learning innovations have been proposed, such the problem-based teaching,^20,21^ mime-based roleplaying simulation training,^22^ and computer-assisted learning,^18^ there is no evidence of the effectiveness of these new methods in real-world settings.

Toward a better basic science/clinical neurology integration, medical students mostly claim that intensive contact with patients with neurologic complaints would be effective to mitigate neurophobia. Longer duration of neurology rotations, more opportunities for extracurricular learning, and expansion of settings of neurologic care (hospital wards, outpatient clinics, and rehabilitation units) could be feasible solutions for this issue.^23^

The temporal comparison showed that, even in a brief period, there were changes in neurophobia in Pará, Brazil. There was an improvement in some categories of perception of neurology/neurosciences of medical students in 2022. Now, the neurologic clinical examination is seen as the second main reason for neurophobia, and a recent trend is favorable to using online resources for better teaching and learning.

A recent review proposed 3 strategies to mitigate neurophobia: (1) establishing a continuum of neurologic education, (2) incorporating active and observed learning, and (3) enhancing socialization into neurology.^24^ In the past 8 years, medical schools in Pará have adopted some of these interventions, such as incorporating neurologists in the neurosciences courses, exposing preclinical students progressively to clinical experiences in neurology, using active learning methods, encouraging students to be involved in neurologic research, and establishing local neurology interest groups. These modifications may explain the measured reduction of neurophobia in our study.

Furthermore, we must consider the impact of the COVID-19 pandemic in Brazil over the past 2 years, which has disrupted the medical education system and harmed undergraduate training.^25^ Due to the pandemic, the forced use of online resources may have influenced medical students' opinions on electronic learning methods.

Our results showed that neurophobia is an active and dynamic issue. Despite preconceived ideas before medical school and other nonmodifiable factors that may partially explain the neurophobic phenomenon, teaching and learning innovations already proposed may be potent drivers to cure neurophobia and promote neurophilia. For that, educational interventions in neurology/neurosciences in low-resource settings should be tested by high-quality trials. In addition, the lack of a core curriculum in neurology for undergraduate medical students in Brazil impairs the possibility of a revolution in neurologic education. The new definition of neurophobia measured by the NeuroQ score will help follow-up on a future reduction of the prevalence of neurophobic medical students caused by teaching and learning solutions.

As limitations, we had a low coverage of assessed students due to the complexity of applying in-person questionnaires during the COVID-19 pandemic. Our strategy to exclude medical students in years 1, 3, and 5 from in-person questionnaires may have reduced the power analysis. The lack of a homogeneous strategy to publicize the online questionnaire between medical schools may have caused a response bias. According to their curricula, the exposure of students to neurosciences/neurology was heterogeneous between medical schools, which may have affected the results. In addition, the study may not reflect the opinions of medical students from other Brazilian regions, and further studies shall test the generalizability of these results.

In conclusion, the prevalence of neurophobia in Brazil was 2-fold to 3-fold higher than that in high-income countries. Unfavorable opinions about neurology tended to increase throughout medical school, but the temporal comparison showed that the impact of neurophobia was slightly lesser in the past years. For a better understanding of this changing issue, surveillance systems for monitoring and tracking neurophobia in medical schools are needed.

## Glossary

COVID-19: coronavirus disease 2019
